# La vida en la frontera: protocol for a prospective study exploring stress and health resiliencies among Mexican-origin individuals living in a US-Mexico border community

**DOI:** 10.1186/s12889-022-14826-x

**Published:** 2022-12-27

**Authors:** Karina R. Duenas, Maia Ingram, Rebecca M. Crocker, Thaddeus W. W. Pace, Jill Guernsey de Zapien, Emma Torres, Scott C. Carvajal

**Affiliations:** 1 Department of Health Promotion Sciences, Arizona Prevention Research Center Zuckerman College of Public Health, University of Arizona, 1295 N. Martin Ave. Tucson, Tucson, AZ 85724 USA; 2 Health Sciences, University of Arizona, Tucson, USA; 3 College of Nursing, Department of Psychiatry, College of Medicine, University of Arizona, Tucson, USA; 4 Department of Psychology, College of Science, University of Arizona, Tucson, USA; 5 Campesinos Sin Fronteras, Somerton, USA

**Keywords:** Ecologic stress, Resilience, Chronic disease risk factors, And Mexican Origin

## Abstract

**Background:**

Mexican-origin adults living near the U.S.-Mexico border experience unique and pervasive social and ecological stressors, including poverty, perceived discrimination, and environmental hazards, potentially contributing to the high burden of chronic disease. However, there is also evidence that residents in high-density Mexican-origin neighborhoods exhibit lower prevalence rates of disease and related mortality than those living in other areas. Understanding the factors that contribute to health resiliencies at the community scale is essential to informing the effective design of health promotion strategies.

**Methods:**

La Vida en la Frontera is a mixed-methods participatory study linking a multi-disciplinary University of Arizona research team with Campesinos Sin Fronteras, a community-based organization founded by community health workers in San Luis, Arizona. This paper describes the current protocol for aims 2 and 3 of this multi-faceted investigation. In aim 2 a cohort of N≈300 will be recruited using door-to-door sampling of neighborhoods in San Luis and Somerton, AZ. Participants will be surveyed and undergo biomarker assessments for indicators of health and chronic stress at three time points across a year length. A subset of this cohort will be invited to participate in aim 3 where they will be interviewed to further understand mechanisms of resilience and wellbeing.

**Discussion:**

This study examines objective and subjective mechanisms of the relationship between stress and health in an ecologically diverse rural community over an extended timeframe and illuminates health disparities affecting residents of this medically underserved community. Findings from this investigation directly impact the participants and community through deepening our understanding of the linkages between individual and community level stress and chronic disease risk. This innovative study utilizes a comprehensive methodology to investigate pathways of stress and chronic disease risk present at individual and community levels. We address multiple public health issues including chronic disease and mental illness risk, health related disparities among Mexican-origin people, and health protective mechanisms and behaviors.

## Rationale

Mexican-origin individuals who live in communities along the U.S.-Mexico border region are embedded in rapidly changing environments replete with intense and pervasive stressors. Psychosocial stressors and associated physiologic responses negatively impact health in the areas of obesity, chronic diseases, and mental illness [1–5]. The most significant health disparities in this population, including diabetes and other metabolic disorders, have known linkages to compromised immune function and heightened inflammatory response [6, 7]. However, despite exposure to economic and social stressors, Mexican-origin individuals and the broader Latino/a population living in the United States (US), have a higher life expectancy and suffer lower rates of many major causes of death than the overall US population [8–11]. The purpose of the La Vida en la Frontera study is to investigate how stress is related to chronic disease risk and to explore sources of resilience within social, cultural, and community-based contexts among Mexican-origin people living along the southwestern Arizona/Sonora border. The study is novel in providing a longitudinal examination of chronic stress on multiple types of biomarkers while quantitatively and qualitatively exploring potential sources of resilience that have protective influences on health.

### Stress, resiliency, and health

While the mechanisms leading from stress to chronic disease are still being discovered, findings show chronic psychological stressors may alter the body’s ability to regulate inflammatory responses and cortisol [12]. Systemic inflammation has been shown to be a risk factor for chronic conditions that are the leading sources of premature mortality in the US and Mexico [13]. Available evidence suggests that disease risk can be transmitted across generations; stressors experienced in past generations can impact a person’s health response today and contribute to stress-induced pathologies [14]. There are other factors that further complicate chronic disease disparities for Latino/as. US-based Latino/a’s age-adjusted poverty rate is roughly 20% (vs 11% and 8% in the US general population and for Non-Hispanic Whites) [15]. Other important social determinants of health status also disproportionately impact Latinos in the US, including that they graduate from high school at the lowest rate in the US (57%) and frequently lack health insurance (42%) [15, 16]. Further, Mexican-origin persons have the lowest health care utilization rates among US groups due to barriers to accessing care, independent of education, income, and insurance status [17].

**Fig. 1:**
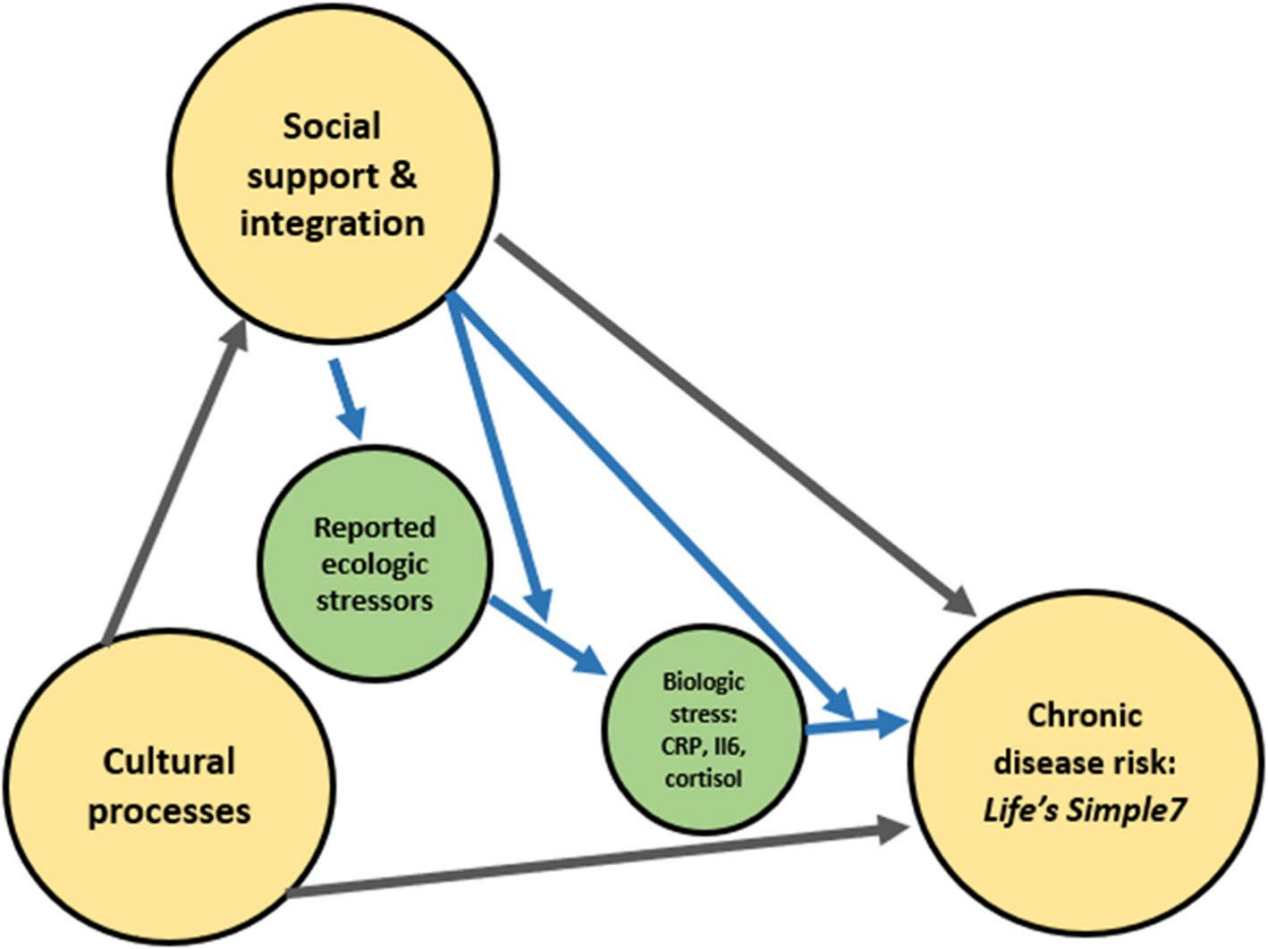
SRM and pathways for reported stressors and inflammatory/stress biomarkers

Despite these disproportionate economic, social, and structural inequities, Latino/as have a longer life expectancy than the overall US population [18]. The Sociocultural Resilience Model (SRM) is one framework to explain this phenomenon [9, 11]. This model identifies important protective factors including social networks, social support, and interrelated coping processes as providing a buffering effect against stress exposure [4, 11]. (Fig. 1). Such protective cultural and social processes may not persist indefinitely, however, and studies report a downward gradient in a broad range of outcomes including wellbeing [3, 10], health-related behavior [19], markers of cumulative stress [20, 21], and obesity [22] according to time spent in the US. Greater exposure to the overarching US culture, acculturative, and assimilative processes may contribute to the decline of the mechanisms which contribute to many of these health advantages [19, 23]. As seen through increasing rates of metabolic disease and discrimination-related stress, [4, 24, 25] the advantages for Mexican-origin individuals may be dissipating as persons spend greater time in the US or non-ethnically dense, enclave, settings. Given the rise in chronic disease and mental illness in both the US and Mexico [13], preserving sociocultural processes and mitigating environmentally related stressors may be critical to preserving healthier outcomes among Mexican-origin persons. Further, evidence for the importance of social support, social networks [12], and other variables and pathways identified by the SRM may aid in effective health promotion strategies and advantages for Mexican-origin individuals, Latino/as more broadly, as well as other groups.

## Methods

La Vida en La Frontera is an integrated mixed methods longitudinal cohort study using a community-based participatory research approach that is exploring how stressors unique to living in a border region contribute to chronic disease risk and investigates sources of resilience within participants’ cultural, social, and binational context. While linguistically assigning gender, we use Latino/a as an all-encompassing term inclusive of all people of Latin American origin. Latino/a is widely used and culturally accepted within the community we are studying. Mexican origin more directly describes our participants as this more closely represents their identity.

The first phase, aligned with Aim 1, qualitatively explored sources of stress and elucidated protective factors [26]. Results from the ethnographically grounded individual interviews informed the development and refinement of measures of stress and resiliency, including items reflecting unique cultural and border contexts. In this protocol paper, we describe aims 2 and 3, the prospective portion of the overall study.

*Aim 2:* To quantitatively assess reported stressors, protective and resiliency factors, biomarkers of inflammation, stress, SARS-CoV-2 immunity, and chronic disease risk in a population-based cohort. We hypothesize that self-reported stress will be positively associated with chronic disease risk at subsequent time points with biomarkers of stress and inflammation acting as mediators and that protective/resilience factors will have direct and moderating effects on biological stress and chronic disease risk.

*Aim 3:* To lead a community-centered appraisal of stress mitigation and reduction intervention strategies, and include an evaluation of the potential adaptation of the Community Health Workers Assisting Latinos Manage Stress and Diabetes (CALMS-D).

intervention. CALMS-D is a Community Health Worker (CHW) led stress management intervention for Latinos with type 2 diabetes comprised of diabetes education and stress management techniques that were developed and tested in the Northwest US [27].

### Setting

Yuma County occupies the outermost region of southwest Arizona neighboring the state of California to the west and Mexico to the south. Two-thirds of the Yuma population is Latino/a and of Mexican origin, and this percentage is much higher in Somerton and San Luis, the two border towns where our study takes place. Agriculture is the leading industry in Yuma County with approximately 38,000 migrant and seasonal farmworkers living in the county. Approximately 15,000 agricultural workers live in the sister city of San Luis Rio Colorado, Mexico, and cross the international border daily to work in the fields. This region's peak agricultural growing season is from September to May when there is a significant influx in the resident population as migrant farmworkers move to the area for work. Like many borderlands, the region includes a shared infrastructure for the economy, health care, education, and cultural, and social ties with the neighboring country, Mexico. The population is not only binational, with existing ties with family, friends, and culture in Mexico, but also transnational, as people regularly cross to access places on both sides of the border for work, recreation, or leisure [28]. It also houses numerous law enforcement and military operations, making it a highly policed and militarized area. Changes in immigration and border policy, distorted portrayals of the border in the media, and increased discrimination and racism impact the environment and local culture. Given the ecologically diverse context, this region is a unique setting to further investigate mechanisms of stress and well-being as it is home to a group of individuals who both experience significant exposure to stressful events but also exhibit resiliencies that protect their health.

### Approach

A central premise of La Vida en La Frontera is that environments are rapidly shifting due to economic and geopolitical shifts [29]. Consequently, developing a research methodology that is attuned to the contexts in which stress and health are embodied necessitates that it be continuously informed by community input. Community-based participatory research (CBPR) is an approach involving key partnerships between researchers and the community under study [30]. Benefits of CBPR include the identification of community-responsive research questions, enhanced data collection and interpretation, and the higher likelihood of using existing infrastructures to sustain interventions beyond the external funding cycle [31]. The academic partner, the Arizona Prevention Research Center (AzPRC) at the University of Arizona, and the primary community partner, Campesinos Sin Fronteras (CSF), co-lead the research project and collaboratively develop and implement all phases of the research study. CSF is a community-based, grassroots organization offering health, housing, and human services to farmworkers and immigrant families along the US/Mexico border in Yuma County. The AzPRC and CSF have collaborated on numerous research and community demonstration projects for over two decades, more than 25 years including the NIOSH-funded farmworker health study that provided some foundations for the present research [25, 32, 33].

CSF was created by former farmworkers to address unmet health and social needs they identified in their community and is an organization composed of community health workers, who are trusted community representatives that provide an array of community services. CHWs are an essential frontline public health workforce that have a close understanding of the community they work in. The CHW workforce has demonstrated effectiveness in addressing health disparities in vulnerable and hard-to-reach communities. CHWs are also valuable members of research partnerships where community and academic groups work together to engage in culturally and community congruent research methodologies to study complex issues present in many high-need regions [34].

### Design

**Fig. 2:**
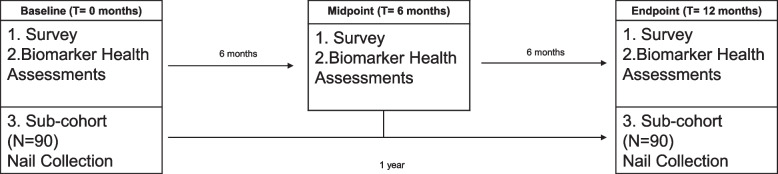
Specific Aim 2 timeline

La Vida en La Frontera is a comprehensive mixed-method study. Aim 2 employs a longitudinal and prospective design that will collect three measures of a cohort of participants (*N* ≈300) across 12 months. Measures include psychosocial and biological indicators of stress, inflammation, and health status, individual and community level protective buffers, social networks and community and cultural processes, and health behaviors at three time points; 6 months apart (Fig. 2). The biological measures will be collected in as minimally invasive methods as possible with information and referrals to respondents to the degree feasible. One of the novel measures will be cortisol collected in nails in a subsample of the larger cohort. Aim 3 will use qualitative methods to identify unique facets of stress and resilience with the Aim 2 cohort sample as we attempt to uncover specific processes that buffer the impact of chronic stress exposure on health. Aim 3 will also qualitatively examine the relevance and potential adaption of the CALMS-D program and other intervention strategies within Southern Yuma County. The study was approved by the UA human subjects’ protection institutional review board (IRB) (2,003,462,440).

### Eligibility

Participants are Mexican-origin adults between the ages of 18 and 70 years old. Inclusion criteria of the study are: 1) live within the cities of San Luis and Somerton Arizona for the majority of the year (i.e. not temporary residents of the region; migrant workers who live in the area for less than half a year as a follow-up would be difficult to not possible) 2) are able to provide informed consent for participation in the study; 3) are be able to complete a 1-h interview style survey in English or Spanish; 4) are able to ambulate to the Campesinos Sin Frontera’s office to complete the biomarker assessments. For the sub- cohort in Aim 2, participants must be able to provide a fingernail or toenail sample with 3 weeks of growth.

### Sampling, recruitment, and retention

Neighborhoods in the towns of San Luis and Somerton, Arizona were randomly selected for door-to-door recruitment. The CHWs provided expertise in neighborhood composition and identified areas in both towns that have a high density of residents (vs commercial areas). The research team identified comparably populated neighborhood segments utilizing town maps and census block data, and randomly selected one in eight for study recruitment.

In teams of two, CHWs visit households in randomly selected neighborhoods and invite eligible individuals to participate. CHWs wear name badges that identify them as representatives from CSF; they also wear T-shirts and ball caps with La Vida en La Frontera, UA, and CSF logos that demonstrate our partnership. Recruitment flyers and door hangers are utilized to introduce and inform the community of the research study’s purpose, incentives, and CSF contact information. Flyers and/ or door hangers with the CHWs’ names are left at the homes of individuals who are not present at the time of canvassing.

We will utilize strategies to continuously engage the participants with the research study including postcards, newsletters, text messages, and phone calls. CHWs will personally contact the participants to confirm or reschedule their next survey appointment two weeks prior and one day before the date. Participants are given a $50 gift card at a local retail outlet determined by the community partner for each visit they complete (1. Survey and Biomarkers and 2. Nail Collection in each time point), and a project labeled stress ball as a retention incentive for their continued participation in the study.

### Sample size

Leveraging the CHWs’ extensive experience in outreach and recruitment, we plan to consent 300 participants for the base cohort via door-to-door recruitment strategies, outlined previously. Anticipating a 33% attrition at the first follow-up and 40% at the second (relative to baseline), we anticipate longitudinal sample sizes of 200 at timepoint 2 and a sample size of 180 at the 2nd follow-up. We estimate N ≈100 sub-cohort of participants for the nail collection portion of the study at baseline and N ≈70 with the follow-up nail sample.

### Aim 2 procedures

In the door-to-door recruitment, CHWs provide complete details about each step of the study to eligible individuals and provide UA IRB approved informed consent to those interested. If the individual is available at the time of door recruitment, they complete the baseline survey battery at that time. If the participant is unavailable, meaning they cannot complete the baseline survey at the time of recruitment, they are scheduled for a future home visit or an office visit within a week to complete the baseline. If no one is home at the time of recruitment the CHWs leave a flyer or a door hanger with information about the study and contact information for CSF. Individuals can contact CSF to inquire about the study and sign up to participate. All participants are scheduled for an in-office visit within one week of completing the baseline survey to complete biomarker data collection.

For the second and third time points, a date is scheduled for approximately 6 months in advance; CHWs inform participants that they will contact them prior to that date and inform them that they are able to reschedule the appointment if they are unavailable at the time. Timepoints two and three follow the same procedure as baseline including a survey battery and biomarker assessments. Participants receive a $50 gift card for participation in each stage of the study, including an additional $50 for participants who are included in the nail collection portion each time they provide a nail sample.

CHWs administer a battery of questionnaires to participants at each time point, using an interview style method in the participant’s preferred language. One CHW gives their full attention to the participant in administering the survey and answering any questions, while the second CHW inputs the participant’s responses on an iPad using RedCap software. This strategy increases CHW engagement with the participant and improves data collection. CHWs also work in teams of two for biomarker data collection in the CSF office. Upon arrival, participants are given a bottle of water and are invited into a private and dedicated office space where the CHW performs the biomarker assessments.

Upon completion of the biomarkers, participants are invited to participate in the sub-cohort nail collection portion of this research project. This is a novel, non-invasive, and culturally sensitive methodology to measure cortisol levels, an indicator of chronic stress in the body. Participants who can provide a sample of finger or toenails with 21 days of growth are eligible to participate in this part of the study. Upon invitation, the CHW screens for eligibility and reviews key aspects of the collection process. They then allow for questions and complete an addendum to the original consent form already signed by the participant. Participants are given a set of nail clippers and instructed to trim and discard their nails and are asked to return in three weeks for specimen collection. Nails are collected at baseline and at the 1-year time point. Nails will be batch analyzed by a collaborating core laboratory using procedures described previously.

Upon completion, the CHWs review the surveys to ensure it is complete and accurate and then upload them directly onto a secure RedCap server. CHWs also work closely with the UA research team’s data manager to monitor and review the data set for missing data or data entry errors. The CHW re-contacts the participant within two weeks to update the survey as needed.

### Aim 2 measures

The survey includes a background questionnaire capturing demographic information, migration history, questions about the neighborhood, and occupation, as well as measures of resilience, mental health, culture and social network, stress, and health. All measures are described in Table 1. Key outcome variables mirror pathways outlined by SRM and include measures of social support and social integration, and stress experiences. Other important variables of interest include measuring resilience through the framework of migration and binational border environments. We have developed a border resilience instrument, informed by Aim 1, to specifically measure factors of resilience present in the border environment.

**Table 1:** Aim 2 measurement summaries

Measure	**Data collected**	**Baseline**	**Midpoint (6 months)**	**Endpoint (1 year)**
Background Questions				
**Demographic**	Gender identity, DOB^a^, Cultural identity, Country of Origin^a^, Years in US^a^, Education, Marital Status, Occupation, Family income, Census participation^a^, Health insurance status	X	X	X
**Migration history**	Country of Origin, Number of Years in the US, Parental Country of Origin,	X	X	X
**Neighborhood questionnaire**	Length of living in Neighborhood and City, Perception of Safety and violence, Binational family, Household composition	X	X	X
**Occupation**	Unemployment, Occupation type, Occupational injury hx	X	X	X
Resilience				
Resilience inventory	Resilience using partial factors from the scale	X	X	X
Border resilience scale	Resilience in a binational border environment	X	X	X
Mental health				
Help seeking questionnaire	Individual willingness to seek and accept help when experiencing a personal or emotional problem	X	X	X
Patient health questionnaire- PHQ-4 and sleep	Symptoms of depression and anxiety including sleep	X	X	X
Mexican American cultural values Scale	Measure of differential Latino cultural expectations	X	X	X
Berkman-syne Social network index	Assesses the type, size, closeness, and frequency of contacts in the participant's current social network	X	X	X
BCISS Stress (adapted)	Stress related to: living in border community, migration, discrimination, COVID-19, medical care, police and border enforcement, and community violence and drug use/trafficking	X	X	X
Health related questionnaires				
BRFSS	Fruit and vegetable consumption	X	X	X
Chronic disease	Self-rated health status, Chronic Disease diagnosis	X	X	X
Health behaviors	Smoking (Tobacco, Marijuana, Vaping), Drug use (Illicit, Prescription)	X	X	X
Medical care utilization	Routine Physical Exam, Routine Laboratory Exams, Mental/Behavioral Health Services	X	X	X
Health assessments				
BMI and adiposity	Height and weight, hip to Waist ratio	X	X	X
Blood Pressure	Systolic, Diastolic, Heart Rate	X	X	X
Dried blood spot	Inflammatory Biomarkers: High sensitivity C-Reactive Protein (CRP), Interleukin-6 (Il6)	X	X	X
Cholesterol	Total Cholesterol, High density lipoprotein, Low density lipoprotein, and triglycerides	X	X	X
Glucose	Random blood sugar test, A1C	X	X	X
Cortisol	Fingernail or Toenail collection	X		X
SARS-Cov-2	Self report information: Exposure, Infection, Vaccination	X	X	X

^a^measured only at baseline

### Biomarkers

Measures include the clinical markers that assess health indicators some of which make up Life's Simple 7 (LS7), an evaluation and indicator of chronic disease risk, identified by the AHA, that can be modified through lifestyle changes and healthy behaviors [35]. Biological markers include body mass index (BMI), blood pressure, cholesterol, glucose, stress, and inflammation markers (i.e., C-reactive protein [CRP] and interleukin-6 [IL-6]), hemoglobin A1c, and SARS-CoV-2 antibodies. To measure makers of inflammation and SARS-CoV-2 antibodies we collect dried blood spots using noninvasive techniques and standard operating procedures approved by UA Institutional Review Board. To measure cortisol, a biological indicator of stress, we collect nails. Nail samples are an alternative and novel measure for chronic retrospective hypothalamic pituitary adrenal axis activation after an encounter with a stressor [36].

We also collect self-report health behavior information from the participant to assess smoking status, nutrition, and activity level. Study partners discussed all the assessments to ensure community appropriateness. At the time of collection, participants receive point of care data for, blood pressure, cholesterol, and random blood glucose meaning that the results of their assessments are provided immediately while at CSF. CHWs then provide informational brochures on CDC recommended guidelines for each of the point of care assessments completed at the visit.

### Aim 2 data analysis plan

Through the data analysis process partners will meet regularly to discuss the proposed analysis and address additional interests raised by study partners. Pending the assessment of the distributional properties of the outcomes, we will utilize multivariable mediational models to test the hypotheses. These models will test total and indirect effects and we will evaluate the overall model fit. We will test the direct pathways from social support/integration measures at wave 1 to reported stress and to LS7 at wave 3. Additionally, higher support/integration will be tested for moderating relations between reported stressors to biologic stress, and from biologic stress to LS7; whereby it is predicted higher levels of support/integration weaken the strength of the paths of reported stress to biologic stress and biologic stress to LS7. Separate models will be done for cortisol.

After analyses, the team will meet to interpret the results and discuss the connection to the community. The team will identify prominent findings, conceptualize the publication agenda, and discuss translation to community benefit and next steps. A key goal for the team is to develop community-level presentations and dissemination of research findings. We will plan community- forums to share findings in the local communities and binational research groups. Local community-based dissemination will be presented in Spanish as it is the most widely used language by participants and in the sampling area more generally.

### Aim 3 procedure

The purpose of Aim 3 is to further investigate how participants with varying levels of self-reported and biologically expressed stress mitigate or cope with stressful situations. This qualitative exploration will be important to subsequent translational work. Some qualitative analysis will be integrated with quantitative findings. As an example, three groups will be identified high stress, low stress, and resilient profiles. Groups will be characterized using a combination of mean standardized scores from the self-report survey and the mean standardized scores of CRP and IL-6 collected at the first two time points. The high-stress groups will have scores in the highest tercile in self-reported stress and the highest tercile in the inflammatory biomarkers. The low-stress group will be in the lowest tercile in reported stress and measured biologic stress/inflammation. The third group will be participants in the tercile with the greatest positive discrepancy in expected inflammation subtracted by their biologically measured inflammation. Selection into this final group in which individuals appear to be effectively managing high levels of stress will take precedence over the low-stress group if participants meet criteria for both.

Sixty participants from Aim 2 will be invited to participate in either an individual interview or a 90-min focus group; this is pending the integration of preliminary analytic results from Aim 2 baseline data. If focus groups are conducted, participants will be separated a priori by the stress and resilient profiles described previously. They will be consented using a UA IRB approved addendum to the consent form previously used in Aim 2. This qualitative aim will further explore perceptions of stress management interventions and techniques including social support, social networks, social conditions, community resources, cross-border social and community connections, and any other prominent findings from Aim 2. Participants will also have with the opportunity to discuss strategies to overcome health, family, and social environmental stressors. Questions will encourage discussion of mechanisms underlying the SRM and how they might promote and preserve health advantages.

### Aim 3 data analysis

The interactions between the facilitator and participants will be recorded and transcribed by professional services. Transcriptions will be analyzed using both a deductive approach based on themes identified in Aims 1 and 2 and components of the SRM and an inductive approach to allow for the identification of new themes. UA researchers and CSF partners will work together to identify prominent themes by reading through the transcriptions and finalizing a code book to guide the coding process. Using Dedoose software, three members of the AzPRC team will double code all data. Throughout the coding process, the partners will engage in an ongoing discussion about emerging themes and together we will examine community congruence. We will identify themes similar across the three groups as well as contrast responses between the sets of groups. For the final data set, we will conduct an analysis of intercoder reliability, and the team will reach a consensus in the case of discrepancy.

## Community benefit and dissemination

An important face of our CBPR approach is to identify community benefits in all aspects of the study. During our bilingual, weekly team meetings, which are completed remotely and include discussion of the key elements of the research process, the CHWs also share their expertise in working with members of the community to inform the development of culturally centered participant engagement, recruitment, and retention. We also assess the community response to the ongoing research activities, which is a critical step to ensuring we are conducting research that is respectful of the community’s cultural and social norms. At least once a month, the academic partners also visit the study location to provide support, engage with participants and have more in-depth discussions with the CHW researchers to identify challenges, opportunities for growth, and successes.

Methods in Aim 2 were developed with input acquired directly from study participants in Aim 1. Specifically, we identified topics of interest and concern to the community including drug use, and social support. We also revised protocols for biomarker assessment and opted to collect samples of finger or toenails versus hair to measure cortisol. We determined that nail samples were more culturally appropriate based on more positive responses from participants interviewed in Aim 1. Before the launch of Aim 2, the protocols, including the survey and biomarkers, were piloted to evaluate implementation and flow. Throughout the development of this study, partners engaged in research-focused interactive workshops on qualitative interviewing, quantitative surveying methods, the biological stress and chronic disease linkage, and biomarker health assessments. In our experience and similar to other research efforts, CHW involvement in the research process increases their capacity to translate skills learned directly to their practice as community leaders and encourages advocacy and the enactment of sustained health promotion practices at individual and systemic levels creating a lasting community resource [37].

All data collected from this study will be used to inform positive change and inform the development of programs to benefit the community and potentially the larger society. Participants will receive immediate results from the immediately interpretable health biomarkers including blood pressure, glucose, and cholesterol (Total, LDL, HDL). While the study’s reports are not diagnostic, they provide valuable health information that an individual may not have access to otherwise. In visiting the CSF offices, participants also are exposed to the vast number of services they have access to in their community.

With respect to peer review publications, results will be interpreted by the research and community partners. Through interactive discussion and writing activities, salient findings will be prepared for publication. All members of the partnership will be encouraged to identify areas of interest to take the lead on manuscript conceptualization. Academic and community researchers will also work in partnerships in developing manuscripts related to some aspects of the study. All members of the team can contribute in the form of discussions or written products.

## Discussion

La Vida en la Frontera is a fully integrated CBPR study that links a UA research team and CSF community researchers to investigate the relationship between stress, health protective mechanisms, and chronic disease risk among Mexican-origin individuals and farmworker families living in a binational border region. This study responds to a need to examine objective and subjective mechanisms of the relationship between stress and health in an ecologically diverse rural community over an extended timeframe and illuminates health disparities affecting residents of this medically underserved community. Further, this investigation leverages the skills of highly experienced CHWs to implement culturally grounded and sensitive approaches to recruitment among a “hard to reach” population, data collection, specimen sampling, and participant retention to ensure lasting community benefit.

From an ethical standpoint, the research and community partners were careful to identify ways in which study participants draw benefit from engaging in the study. Participants receive important health information, such as blood pressure, BMI, cholesterol, glucose, and waist and hip measurements which they otherwise may lack due to barriers to access to essential and preventative medical care services. Individuals are given educational information and materials about health biomarkers during their visits, which include CDC guidelines for health markers. Participants are encouraged to follow up with a medical provider to discuss their health information when possible. If the participant does not have a primary medical provider, the CHWs connect them with relevant services available in the community. Health literacy through increased awareness of self-health status empowers individuals to take intentional steps towards modifying behaviors and improving health. Additionally, via regular visits to the CSF offices over the course of the study, participants have the opportunity to interface with other CSF program offerings. This has the potential to engage both the individual and CSF in new community partnerships and increase their networks.

Findings from this investigation directly impact the participants and community through deepening our understanding of the linkages between individual and community level stress and chronic disease risk. Participant engagement and community benefit is a fundamental interest of our research team and results from this study will be presented directly within community settings.

## Conclusion

Mexican-origin people living in US-Mexico border regions are exposed to high ecologic stressors and health-related disparities which can negatively impact their health and overall increase their risk for the development of disease [32]. Health disparities in the US disproportionately affect ethnic minorities including Latino/as and contribute to increased risk for the development of chronic disease, mental illness, and overall poor health outcomes [38]. Overall, chronic disease prevalence has increased in the US with roughly 30% of the adult population having been diagnosed with more than one condition [39]. However, Latino/as also exhibit protective mechanisms which buffer the effect of stress-disease pathways [4]. While there are prior studies that have examined the relationship between stress and health outcomes, we aim to also examine mechanisms leading to health resiliencies in a medically underserved population [40, 41]. This study is innovative as it employs a comprehensive mixed methods approach to investigate pathways of stress exposure and chronic disease risk including mediating protective characteristics present in individual and community level characteristics. Further, this proposed study employs a community-based approach to study these relationships across an extended time period, providing novel and important information. This study addresses multiple public health issues including chronic disease and mental illness risk, health-related disparities among Mexican-origin people, and health protective mechanisms and behaviors. We use innovative and community-based approaches and have developed and implemented methodologies that benefit the community throughout the implementation of the research. Results from this study will directly inform 1) community-level interventions, 2) community-level policy change, and 3) individual awareness of health information for participants involved in the study.

## Data Availability

Not applicable.

## Abbreviations

US: Unites States
SRM: Sociocultural Resilience Model
CALMS-D: Community Health Workers Assisting Latinos Manage Stress and Diabetes CHW: Community Health Worker
CBPR: Community-based participatory research
AzPRC: Arizona Prevention Research Center
CSF: Campesinos Sin Fronteras
IRB: Institutional review board
LS7: Life's Simple 7
BMI: Body mass index
CRP: C-reactive protein
IL-6: Interleukin-6
LDL: Low density lipoprotein
HDL: High density lipoprotein
NIMHD: National Institute on Minority Health and Health Disparities
